# Legal Tetrahydrocannabinol in the US—Regulatory Loopholes

**DOI:** 10.1001/jamahealthforum.2026.1385

**Published:** 2026-05-01

**Authors:** Grant C. Nippler, Jennifer L. Pearson, Abigail S. Friedman

**Affiliations:** Department of Health Policy and Management, Yale School of Public Health, New Haven, Connecticut (Nippler, Friedman); Department of Health Behavior, Policy, and Administration Sciences, Reno School of Public Health, University of Nevada, Reno (Pearson).

Although federal law bars cannabis sales, nonmedical products containing intoxicating levels of tetrahydrocannabinol (THC)—cannabis’ primary psychoactive component—can be sold legally in most US states. Beyond states legalizing adult-use cannabis sales, the 2018 Farm Bill’s (Pub L No. 115-334) federal legalization of hemp (defined as any part of a cannabis plant with Δ9-THC content of up to 0.3% of dry weight [ie, after moisture is removed]) created 2 loopholes: Manufacturers can sell intoxicating, hemp-derived products by including higher concentrations of other hemp-derived cannabinoids (eg, Δ8-THC, Δ0-THC, and Δ10-THC, which have varying potencies) or by producing greater dry-weight products to legally deliver more Δ9-THC.^1^ Consequently, many consumers can legally purchase high-dose THC products without the safeguards that constrain legal cannabis markets (eg, product testing and minimum age restrictions).

The Continuing Appropriations, Agriculture, Legislative Branch, Military Construction and Veterans Affairs, and Extensions Act of 2026 (HR 5371) establishes more restrictive limits on hemp-derived products, capping the final product’s total THC to “0.4 milligrams combined total per container” of both THC and “any other cannabinoids that have similar effects.”^2^ However, those provisions do not go into effect until November 12, 2026, and may never be implemented if lobbying and lawsuits are successful.^3^ In the interim, regulatory gaps for hemp-derived THC products raise practical concerns, amplified by executive action directing the US attorney general to reschedule cannabis from Schedule I to Schedule III^4^ without explicitly assigning regulatory authority.

## THC Regulation in the US

As of October 2025, US consumers can legally purchase nonmedical THC products through regulated adult-use cannabis markets (23 states with retail sales in progress), minimally regulated markets for hemp-derived THC (24 states, 6 of which allow adult use), or markets that allow low-potency hemp-derived THC products, where consumers may achieve higher doses by consuming multiple servings (12 states, 8 allowing adult use).^5^ Remaining states require no detectable THC in consumable hemp products (Idaho), prohibit its addition to food and drinks (Mississippi, North Dakota, and New Hampshire), or have evolving or contested hemp-derived THC policies (Massachusetts, Missouri, Nebraska, New Jersey, and Ohio).^5^

While labeling inaccuracies exist in regulated and unregulated markets, products in unregulated contexts routinely contain more THC than advertised,^6^ and most states do not require THC warning labels. Thus, patients may be misinformed or entirely unaware of their true exposure, potentially compromising clinical risk assessment and treatment decisions, given research linking cannabinoids to complications from anesthesia and a growing list of drug interactions.^7,8^

Current regulation also fails to protect youths: Of the 24 states that allow intoxicating hemp-derived THC products, 10 lack age restrictions and 14 fail to regulate associated advertising.^5^ Indeed, hemp-derived products rarely face the same packaging and advertising regulations as cannabis in legal adult-use states, despite risks of youth use and pediatric poisoning.^9^

## Insights From Tobacco and Alcohol Regulation

The aforementioned regulatory failures echo challenges in US tobacco regulation. The 2009 Family Smoking Prevention and Tobacco Control Act (Pub L No. 111-31) gave the US Food and Drug Administration (FDA) regulatory authority over cigarettes, several forms of loose tobacco, and smokeless tobacco, empowering the FDA to extend its jurisdiction to any tobacco-derived product intended for human consumption. Nicotine vaping companies thus avoided federal regulation for years before the FDA deemed all tobacco products under its authority. Subsequently, the industry shifted toward synthetic nicotine, a loophole Congress closed in 2022 by updating the definition of tobacco products to cover products containing nicotine where the latter was made or derived from nontobacco sources. Parallels with cannabis regulation are evident: Facing product-specific policies, firms developed new nicotine products. Facing plant-specific policies, firms developed intoxicating THC products derived from the less heavily regulated plant.

Alcohol offers another instructive counterexample. Because alcohol-related impairment stems from a single compound (ethanol), federal law avoids the aforementioned loopholes by regulating alcoholic beverages based on ethanol content (ie, alcohol by volume), regardless of the material used for fermentation.

Comprising a diverse and evolving family of compounds and analogs, intoxicating cannabinoids present a more complex case. Setting aside fully synthetic cannabinoids—scheduled separately under the Controlled Substances Act and not subject to plant-based regulation (21 USC §812)—this chemical diversity makes plant-based regulation especially vulnerable to loopholes, particularly where firms can develop high-potency products from the less-restricted plant (eg, via selective breeding of cultivars or reformulation of products toward higher dry weights). Regulating the final product’s total intoxicating cannabinoid content avoids such workarounds.

## A Concrete Regulatory Approach to Cannabinoids

Effectively regulating psychoactive cannabinoids requires assigning responsibility to a specific government agency charged with developing evidence-based standards and protocols for testing, labeling, auditing, and enforcement. Expertise and jurisdiction make the FDA the logical choice for this role: the FDA already reviews cannabinoid pharmaceuticals and regulates foods (a common mode of delivery for hemp-derived THC). The impending rescheduling of cannabis from Schedule I to Schedule III makes this pressing: Congress needs to amend the Federal Food, Drug, and Cosmetic Act (21 USC §301 et seq) to place rescheduled and descheduled cannabinoids under FDA purview, funding or requiring user fees to fund associated offices and research accordingly.

This research funding is critical. Targeting total cannabinoid content in consumable products will close loopholes created by arbitrary distinctions between hemp- and cannabis-derived compounds but requires several innovations. First, uniform testing methods are needed to reliably establish potency. This is a prerequisite for vital research into the risks from different levels of cannabinoid exposure to determine appropriate thresholds for retail products, accounting for formulation and compound (eg, due to stronger vs weaker binding to cannabinoid receptors in Δ9-THC vs Δ8-THC, or contaminants in semisynthetic cannabinoids). Alongside basic science, such research should engage existing state-licensed laboratories to identify reliable, scalable processes and leverage population health data and natural experiments to assess risks related to formulation and behavior (eg, mode of use, consuming multiple servings). Resulting evidence-based standards, paired with uniform regulatory standards for potency labeling, child-resistant packaging, and minimum age restrictions, are critical for consumer protection.^10^

Finally, strategies to address the implications of cannabinoid use for patient care are urgently needed. As legal considerations and stigma may reduce patient disclosure to clinicians, informing patients about potential drug interactions should follow the example of alcohol: known, clinically significant interactions with cannabinoids should be stated on the drug’s label and in the FDALabel database. Where such interactions have not been established, a separate warning could note this fact; in addition, the FDA might prioritize assessment of potential contraindications for medications known to interact with FDA-approved cannabinoid-based drugs (eg, dronabinol and Epidiolex, a prescribed cannabidiol oral solution). Requiring pharmacists to reiterate these risks and uncertainty upon dispensing medications would help patients make more informed decisions around concurrent cannabinoid and prescription medication use, without requiring disclosure.

Addressing the aforementioned challenges requires a concerted effort between policymakers, regulators, researchers, and clinicians to remove regulatory loopholes, establish evidence-based standards, and inform associated policies and protocols. The first necessary step is for Congress to assign regulatory responsibility for non–Schedule I cannabinoids to the FDA—focusing on chemicals of concern regardless of plant source—and to allocate critical funding to establish standardized testing procedures. Thereafter, research can assess risks from different cannabinoids, potencies, and product types to inform evidence-based policies that protect population health.
